# Lower mortality risk in *APOE4* carriers with normal cognitive ageing

**DOI:** 10.1038/s41598-023-41078-5

**Published:** 2023-09-12

**Authors:** Elizabeth Pirraglia, Lidia Glodzik, Yongzhao Shao

**Affiliations:** 1 Department of Population Health, New York University Grossman School of Medicine, New York, NY USA; 2 Department of Radiology, Weill Cornell Medicine, Brain Health Imaging Institute, New York, NY USA

**Keywords:** Risk factors, Alzheimer's disease, Outcomes research

## Abstract

Abnormal cognitive ageing, including dementia, poses serious challenges to health and social systems in ageing populations. As such, characterizing factors associated with abnormal cognitive ageing and developing needed preventive measures are of great importance. The ε4 allele of the Apolipoprotein E gene (*APOE4*) is a well-known genetic risk factor for late-onset Alzheimer’s disease. *APOE4* carriers are also at elevated risk of cardiovascular diseases which are associated with increased risk of cognitive impairment. On the other hand, *APOE4* is known to be associated with reduced risk of multiple common types of cancer—a major age-related disease and leading cause of mortality. We conducted the first-ever study of *APOE4’s* opposing effects on cognitive decline and mortality using competing risk models considering two types of death—death with high-amounts versus low-amounts of autopsy-assessed Alzheimer’s neuropathology. We observed that *APOE4* was associated with decreased mortality risk in people who died with low amounts of Alzheimer’s-type neuropathology, but *APOE4* was associated with increased mortality risk in people who died with high amounts of Alzheimer’s-type neuropathology, a major risk factor of cognitive impairment. Possible preventive measures of abnormal cognitive ageing are also discussed.

## Introduction

Abnormal cognitive ageing, including dementia, in elderly populations poses a serious challenge to health and social systems around the globe. As such, characterizing factors associated with age-related cognitive decline as well as developing needed preventive measures are of great importance. The ε4 allele of the Apolipoprotein E gene (*APOE4*) is a major risk factor for abnormal cognitive ageing, as it is the strongest genetic risk factor for late-onset Alzheimer’s disease^1^. Alzheimer’s disease is the most common cause of dementia, accounting for approximately 60 to 80% of dementia cases which are a devastating type of abnormal cognitive ageing^1^. Carriers of the ε4 allele (*APOE4* carriers) are particularly vulnerable to developing dementia and to the accumulation of amyloid plaques and neurofibrillary tangles—the hallmarks of Alzheimer’s disease (AD) neuropathology^2,3^. This vulnerability is associated with synaptic degeneration, synaptic loss, and neuron death which accelerate the process of abnormal cognitive ageing and adversely affect both quality of life and mortality. While some degree of memory loss, as well as the accumulation of low amounts of plaques and tangles in the brain, are part of normal cognitive ageing, dementia is not a normal part of the cognitive ageing process. Beyond memory loss, symptoms of Alzheimer’s type dementia can include behavioral changes, delusions, paranoia, aggression, and depression^4^. These symptoms have devastating effects on quality of life for patients as well as their families. However, some promising preventive measures have emerged in recent years that might be further optimized to effectively reduce the risk of age-related cognitive decline among vulnerable subpopulations such as *APOE4* carriers. In addition to the adverse impact on cognitive ageing and quality of life, AD is one of the leading causes of death in people 65 years and older^1,5,6^. *APOE4* is more prevalent among people with AD (58% versus 23% among non-demented)^7,8^, however, not all *APOE4* carriers develop AD or experience abnormal cognitive ageing even at advanced ages (e.g., centenarians).

The association of *APOE4* with abnormal cognitive ageing and mortality in elderly people is complicated from the opposing effects of *APOE4* on the top two causes of death globally^9,10^ – cardiovascular diseases (CVD) and cancer^11^. On one hand, many studies have reported that *APOE4* is associated with elevated risk of CVD^12–15^. Furthermore, as the top leading cause of death, CVD can also have a detrimental impact on cognitive ageing and increased risk of accumulating AD neuropathology, particularly among *APOE4* carriers^16–18^. On the other hand, numerous studies have reported that *APOE4* is associated with reduced risk of several common types of cancer^19–21^ – the second leading cause of death globally^9^. As such, there is a high proportion of *APOE4* carriers with normal cognitive ageing at the late-stages of life^8^. These opposing effects indicate substantial heterogeneity in cognitive ageing and mortality risk among *APOE4* carriers.

Competing risk survival analysis is well-suited for analyzing heterogeneous associations between *APOE4* and different causes of cognitive decline or types of death^22^. In a competing risk survival model, cause-specific hazard ratios are estimated separately for each type of death^23,24^. In the presence of opposing effects, the cause-specific hazard ratios can offer useful insights and more meaningful interpretations of the data than the overall hazard ratio from an all-cause survival model.

While many studies have analyzed the impact of *APOE4* on all-cause mortality^25–28^, ours is the first to thoroughly investigate the heterogeneous associations and opposing effects of *APOE4* on two competing types of mortality risk by accounting for the low versus high amounts of autopsy-assessed AD neuropathology in the brain at death. The National Alzheimer’s Coordinating Center (NACC) has a large collection of brain autopsy data with state-of-the-art quantifications of AD neuropathology, providing a unique opportunity to examine the heterogeneous impact of *APOE4* on mortality risk. Using the NACC data, we examined competing types of deaths accounting for the amount of autopsy-assessed AD neuropathology present at the time of death. Not only is the autopsy assessment of AD neuropathology widely considered to be the gold-standard diagnosis of AD^29–31^, it also gives an accurate measurement of neuropathology presence at the time of death, whereas a clinical diagnosis of AD would have been made some time before death.

The amount of AD neuropathology present in the brain is known to be correlated to the risk of synaptic impairment, neuronal loss, accelerated cognitive decline and elevated risk of mortality. Our primary aim in this project was to compare the impact of *APOE4* on mortality risk for two competing types of deaths: death with low amounts of AD neuropathology versus death with high amounts of AD neuropathology, and to discuss potential preventive measures for developing high amounts of neuropathology among *APOE4* carriers. We used competing risk survival analysis to compare the mortality risks for these two competing types of death assessed at autopsy. To reduce potential selection bias related to consent for brain autopsy donation, our competing risk survival analysis incorporated an assessment of autopsy propensity as discussed in detail in the Methods section. Briefly, our primary analysis was modeled in two steps. In the first step, a logistic model was developed to predict autopsy propensity using subjects who had died. This model was then used to derive autopsy propensity scores for all subjects (living and dead). In the second step, percentile categories of the autopsy propensity scores were incorporated as model strata into stratified competing risk survival models of people who died with the two types of death as competing risks. These analyses also included living subjects as censored observations. We used both the cause-specific hazards model (CSH)^24^ and the Fine and Gray subdistribution hazard model^23^ to assess the heterogeneous mortality risk associated with *APOE4*^22^.

## Results

### Sample characteristics

This study used the National Alzheimer’s Coordinating Center (NACC) data repository which is composed of data submitted from 45 NIA Alzheimer’s Disease Research Centers (ADRC’s) from across the US. As of March 2023, the NACC database included 45,998 individuals recruited since 2005. More details about sampling and inclusion/exclusion criteria are described in the Methods section.

The demographic and clinical characteristics for subjects in the two competing risk groups and censored subjects are summarized in Table 1. As shown in Table 1, the competing risk group with low amounts of AD neuropathology at death (DeadLowADnp) had younger baseline ages, a lower proportion of females, less cognitive impairment at baseline, and shorter study follow-up time compared to the competing risk group with high amounts of AD neuropathology (DeadHighADnp). Censored subjects had younger baseline ages, higher proportions of females and people who identified as non-White or Hispanic, as well as less cognitive impairment at baseline, compared to both the DeadLowADnp and DeadHighADnp groups. Censored subjects had longer study follow-up times compared to the DeadLowADnp group but had similar follow-up times to the DeadHighADnp group.

**Table 1:** Demographics and clinical characteristics for study groups from the NACC cohort.

	DeadLowADnp	DeadHighADnp	Censored
	(n = 1,889)	(n = 3,857)	(n = 24,681)
Age at baseline			
Mean [SD]	74.44 [12.00]	76.30 [10.44]^a^	70.21 [9.99]^a,b^
Median (IQR)	76 (66–84)	78 (70–84)	71 (65–77)
Sex, No. (%)			
Female	827 (44%)	1843 (48%)^a^	14,780 (60%)^a,b^
Male	1062 (56%)	2014 (52%)	9901 (40%)
Race, No. (%)			
White	1765 (93%)	3600 (93%)	19,417 (79%)^a,b^
Black or African American	52 (3%)	138 (4%)	3219 (13%)
Native American/Alaskan	1 (< 1%)	4 (< 1%)	140 (1%)
Hawaiian/Pacific Islander	3 (< 1%)	0 (< 1%)	17 (< 1%)
Asian	19 (1%)	24 (1%)	739 (3%)
Multiracial	35 (2%)	70 (2%)	822 (3%)
Unknown/missing	14 (1%)	21 (1%)	327 (1%)
Ethnicity, No. (%)			
Hispanic	57 (3%)	144 (4%)	2116 (9%)^a,b^
Non-Hispanic	1824 (97%)	3693 (96%)	22,474 (91%)
Unknown/missing	8 (< 1%)	20 (1%)	91 (< 1%)
Education (years)			
Mean [SD]	15.50 [3.1]	15.32 [3.15]	15.35 [3.38]
Median (IQR)	16 (13–18)	16 (13–18)	16 (13–18)
Baseline diagnosis, No. (%)			
Cognitively unimpaired	607 (32%)	584 (15%)^a^	11,890 (48%)^a,b^
Cog. imp (not demented)	364 (19%)	661 (17%)	6985 (28%)
Non-AD dementia	609 (32%)	371 (10%)	1151 (5%)
AD	309 (16%)	2241 (58%)	4655 (19%)
Baseline global CDR, No. (%)			
No impairment	639 (34%)	581 (15%)^a^	11,962 (48%)^a,b^
Questionable impairment	591 (31%)	1263 (33%)	9174 (37%)
Mild impairment	364 (19%)	1083 (28%)	2668 (11%)
Moderate impairment	171 (9%)	481 (12%)	722 (3%)
Severe impairment	124 (7%)	449 (12%)	155 (1%)
Follow-up time (years)			
Mean [SD]	3.48 [3.27]	3.80 [3.15]^a^	3.85 [3.85]^a^
Median (IQR)	3 (1–5)	3 (1–6)	3 (1–6)
Age at death			
Mean [SD]	79.33 [12.76]	81.76 [10.7]	NA
Median (IQR)	81 (70–90)	83 (74–90)	

DeadLowADnp = the competing risk group of individuals who died with low amounts of Alzheimer’s neuropathology.

DeadHighADnp = the competing risk group of individuals who died with high amounts of Alzheimer’s neuropathology.

Censored = all individuals who were not indicated to be dead.

SD = standard deviation.

IQR = Interquartile range.

Cog. imp = cognitively impairment.

AD = Alzheimer’s disease.

CDR = Clinical Dementia Rating (CDR®) Dementia Staging Instrument score.

^a^Statistically significant difference (at *p* < .017 with Bonferroni correction) from the DeadLowADnp group.

^b^Statistically significant difference (at *p* < .017 with Bonferroni correction) from the DeadHighADnp group.

The *APOE* allele distributions for subjects in the two competing risk groups and censored subjects are shown in Table 2. These *APOE4* allele distributions are very similar to reported distributions from studies of the general population. In particular, the *APOE4* prevalence in the DeadLowADnp group of 21% was similar to the estimated 23% in non-demented elderly populations^8^, and the 56% prevalence among the DeadHighADnp group was similar to the average population estimate of 58% for people with autopsy-assessed AD^7^. The *APOE4* prevalence in the censored subjects was 40%, which was higher than the DeadLowADnp and lower than the DeadHighADnp group. This was not surprising since this group contains a mix of subjects, some of whom will eventually die with high amounts of AD neuropathology and others with low amounts.

**Table 2:** *APOE* allele distributions for study groups from the NACC cohort.

	DeadLowADnp	DeadHighADnp	Censored
	(n = 1,889)	(n = 3857)	(n = 24,681)
*APOE* allele, no. (%)			
ε3/ε3	1183 (63%)	1545 (40%)	12,460 (50%)
ε2/ε3	287 (15%)	162 (4%)	2357 (10%)
ε2/ε2	18 (1%)	7 (< 1%)	110 (< 1%)
ε2/ε4	33 (2%)	127 (3%)	601 (2%)
ε4/ε4	25 (1%)	462 (12%)	1539 (6%)
ε3/ε4	343 (18%)	1554 (40%)	7614 (31%)
*APOE4* carriers	401 (21%)	2143 (56%)^a^	9754 (40%)^a,b^

DeadLowADnp = the competing risk group of individuals who died with low amounts of Alzheimer’s neuropathology.

DeadHighADnp = the competing risk group of individuals who died with high amounts of Alzheimer’s neuropathology.

Censored = all individuals who were not indicated to be dead.

*APOE4* carriers = individuals with at least one ε4 allele (i.e., ε2/ε4, ε3/ε4, and ε4/ε4).

^a^Statistically significant difference (at *p* < .017 with Bonferroni correction) from the DeadLowADnp group.

^b^Statistically significant difference (at *p* < .017 with Bonferroni correction) from the DeadHighADnp group.

### Autopsy propensity score model

As the first step in our primary analysis, a logistic regression model was used to derive autopsy propensity scores to reduce potential bias related to consent for brain autopsy donation. Five variables were selected to be included in the model using backward stepwise selection with cross-validation. Having a self-identified race as White (compared to non-White) was the strongest predictor of autopsy participation (OR = 2.73, 95% CI 2.38–3.12), followed by living in a senior community (independent, assisted living, or nursing facility vs. private residence, OR = 1.68, 95% CI 1.49–1.89), participation in a research study (OR = 1.48, 95% CI 1.35–1.63), higher Clinical Dementia Rating (CDR®) Dementia Staging Instrument sum of boxes (OR = 1.44, 95% CI 1.37–1.51), and years of education (OR = 1.26, 95% CI 1.20–1.31). *APOE4* was not a significant predictor of autopsy propensity univariately nor as a covariate if added to the final propensity-score model. This logistic regression model was used to predict autopsy propensity scores for all subjects in our study. The predicted scores were then broken into discrete percentile categories. The second step in our primary analysis was to incorporate these autopsy propensity score percentile categories as strata in the stratified competing risk survival models.

### Competing risk survival models

**Figure 1:**
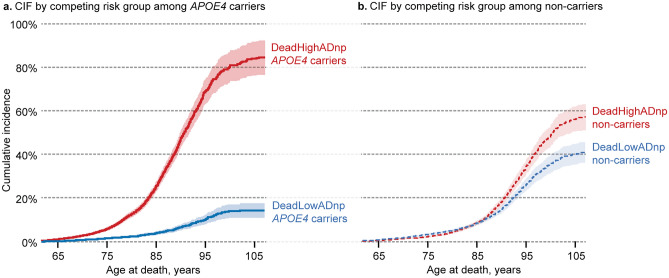
The cumulative incidence function (CIF) plot for the competing risk groups is shown for *APOE4* carriers in panel (**a**), and non-carriers in panel (**b**). The cumulative incidence (as a proportion) is on the y-axis and the age at death (in years) is on the x-axis. *APOE4* carriers who died with high amounts of Alzheimer’s lesions (DeadHighADnp *APOE4* carriers, red solid line) had the highest incidence of death, followed by non-carriers (DeadHighADnp non-carriers and DeadLowADnp non-carriers, red and blue dashed lines, respectively). The lowest incidence of death was among *APOE4* carriers who died with low amounts of AD neuropathology (DeadLowADnp *APOE4* carriers, blue solid line). The 95% confidence intervals (shaded areas) of the CIF—calculated by the Fine and Gray method—show no overlap in the cumulative incidences of death for the competing risks of death among *APOE4* carriers and non-carriers.

**Figure 2:**
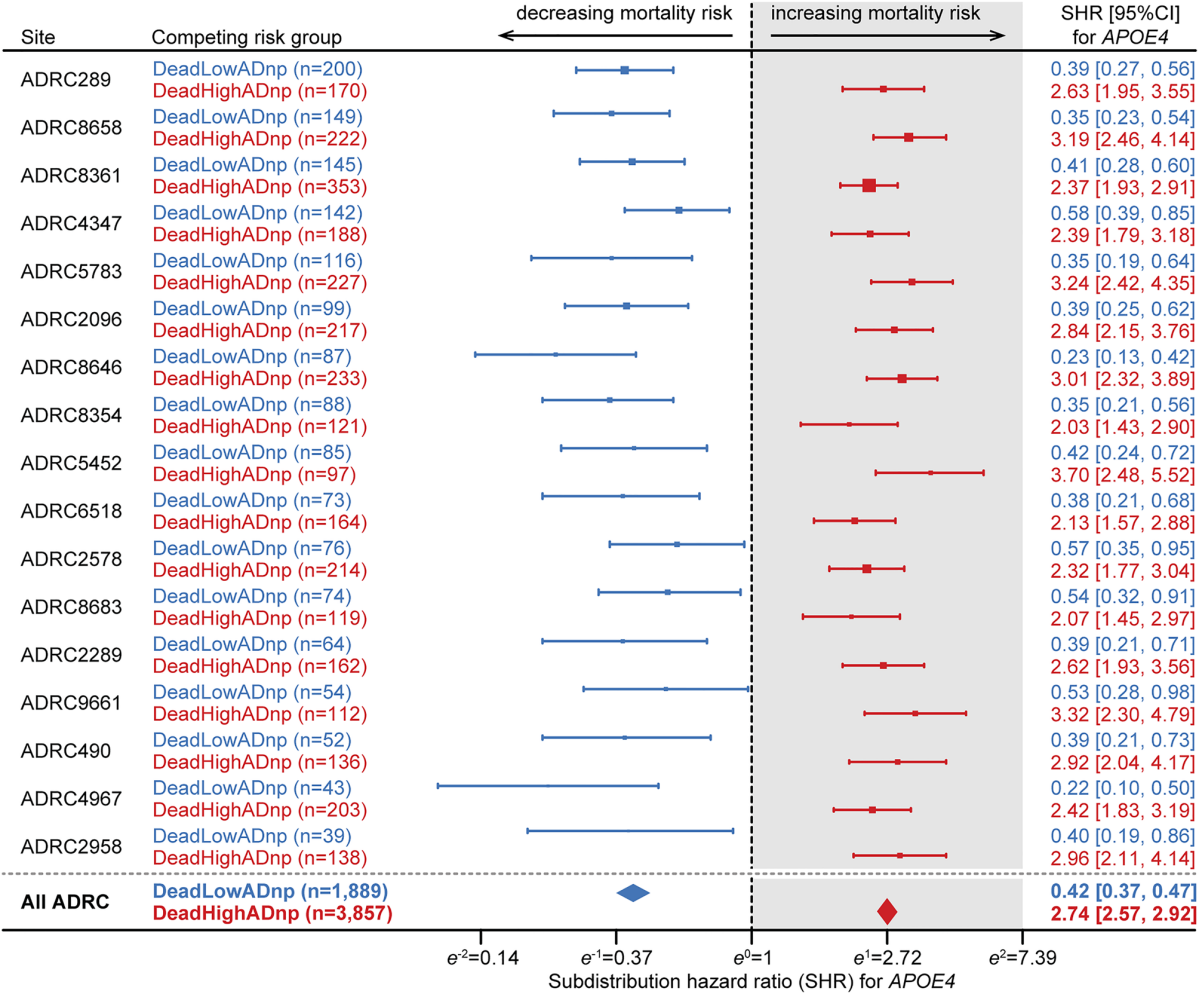
Forest plots of univariate Fine and Gray competing risk survival analyses are shown in the 17 Alzheimer’s Disease Research Centers (ADRC) that had at least 165 autopsied people (representing > 80% of the autopsy sample). The colored horizontal bars represent the estimated 95% confidence interval of the subdistribution hazard ratio (SHR) for *APOE4* on mortality risk, with the box in the middle at the estimated SHR scaled to the size of the sub-sample. The dashed vertical line is at exp(β = 0) = 1, with decreasing mortality risk on the left and increasing mortality risk on the right. The SHR estimates for the competing risk group of death with low amounts of AD neuropathology (DeadLowADnp, shown in blue) are all to the left of the dashed vertical line, indicating decreased mortality risk was associated with *APOE4* for this competing risk group in all 17 ADRC. The SHR estimates for the competing risk group of death with high amounts of AD neuropathology (DeadHighADnp, shown in red) are all to the right of the dashed vertical line, indicating increased mortality risk was associated with *APOE4* for this competing risk group in all 17 ADRC. The SHR for *APOE4* on mortality risk from the univariate competing risk model including all ADRC is shown in bold at the bottom of the figure.

The cumulative incidence function (CIF) plot from the Fine and Gray subdistribution competing risk model (Fig. 1a,b) showed the lowest incidence of death for *APOE4* carriers in the DeadLowADnp competing risk group compared to all other groups. In contrast, *APOE4* carriers in the DeadHighADnp group had the highest incidence of death. In the Fine and Gray competing risk survival model, the 95% confidence interval (CI) of the subdistribution hazard ratio (SHR) for *APOE4* was below one in people who died with low amounts of AD neuropathology (DeadLowADnp). This indicated that *APOE4* carriers in the DeadLowADnp competing risk group had decreased mortality risk (SHR = 0.42, 95% CI 0.37–0.47) compared to non-carriers (Fig. 2). In contrast, the 95% CI of the SHR for *APOE4* was above one in people who died with high amounts of AD neuropathology (DeadHighADnp), indicating that *APOE4* carriers in the DeadHighADnp competing risk group had increased mortality risk (SHR = 2.74, 95% CI 2.57–2.92) compared to non-carriers (Fig. 2). The pattern of the cause-specific hazard ratio (CSHR) and the SHR from the Fine and Gray competing risk survival model were consistent for both *APOE4* carriers in the DeadLowADnp competing risk group (CSHR = 0.53, 95% CI 0.48–0.60) and in the DeadHighADnp competing risk group (CSHR = 2.67, 95% CI 2.50–2.84). Furthermore, in multivariable models (including covariates as well as autopsy propensity scores as a stratifying variable) *APOE4* was likewise associated with decreased mortality risk in the DeadLowADnp competing risk group (SHR = 0.33, 95% CI 0.29–0.37; CSHR = 0.40, 95% CI 0.36–0.45), and with increased mortality risk in the DeadHighADnp competing risk group (SHR = 2.22, 95% CI 2.06–2.39; CSHR = 1.68, 95% CI 1.57–1.80).

Significant baseline covariates in the competing risk survival models included sex, functional independence, cognitive impairment, and global CDR score. In both competing risk groups, reduced mortality risk was associated with females and being functionally independent, cognitively unimpaired, or having lower global CDR scores. There were no significant three-way or higher-order interactions in the multivariable competing risk models. The β coefficients and p-values for the multivariable models including all two-way interactions that had significant (*p* < 0.05) CSHR and SHR are shown in Table 3. In particular, in the DeadHighADnp competing risk group, there were significant two-way interactions of *APOE4* with age (SHR = 0.977, 95% CI 0.966–0.989; CSHR = 0.986, 95% CI 0.979–0.993) and with female sex (SHR = 0.850, 95% CI 0.739–0.977; CSHR = 0.744, 95% CI 0.652–0.849). In the DeadLowADnp competing risk group, there was a significant interaction between *APOE4* and cognitive impairment (SHR = 0.401, 95% CI 0.314–0.512; CSHR = 0.433, 95% CI 0.337–0.556). In sensitivity analysis, the direction of the main findings for *APOE4* remained the same with or without including censored subjects, adjusting for covariates, or using the autopsy propensity scores as a stratifying variable.

**Table 3:** Multivariable competing risk models with main effects and significant 2-way interaction terms.

Model term	DeadLowADnp				DeadHighADnp			
	*CSHR model*		*SHR model*		*CSHR model*		*SHR model*	
	β coef	*p*-value	β coef	*p*-value	β coef	*p*-value	β coef	*p*-value
*APOE4* (carrier = 1)	− 0.264	0.015	− 0.414	< 0.001	1.74	< 0.001	2.624	< 0.001
Age	− 0.187	< 0.001	− 0.001	0.86	− 0.197	< 0.001	0.023	< 0.001
Fun. indep (yes = 1)	− 1.055	< 0.001	− 0.737	< 0.001	− 0.472	< 0.001	− 0.105	0.075
Cog. imp (yes = 1)	9.114	< 0.001	6.734	< 0.001	11.32	< 0.001	6.682	< 0.001
Global CDR	0.285	< 0.001	− 0.072	0.17	0.774	< 0.001	0.41	< 0.001
Sex (female = 1)	− 0.492	< 0.001	− 0.412	< 0.001	− 0.185	< 0.001	− 0.076	0.14
Age x cog. imp	− 0.107	< 0.001	− 0.082	< 0.001	− 0.126	< 0.001	− 0.072	< 0.001
*APOE4* x age					− 0.014	< 0.001	− 0.023	< 0.001
*APOE4* x sex					− 0.295	< 0.001	− 0.163	0.022
*APOE4* x cog. imp	− 0.838	< 0.001	− 0.914	< 0.001				

DeadLowADnp = the competing risk group of individuals who died with low amounts of Alzheimer’s neuropathology.

DeadHighADnp = the competing risk group of individuals who died with high amounts of Alzheimer’s neuropathology.

Cog. imp = cognitively impairment.

Fun. indep = functional independence.

CDR = Clinical Dementia Rating (CDR®) Dementia Staging Instrument score.

*APOE4* carriers = individuals with at least one ε4 allele (i.e., ε2/ε4, ε3/ε4, and ε4/ε4).

Competing risk survival models were conducted within each individual Alzheimer’s Disease Research Centers (ADRC’s) from the NACC data repository, to check for stability and consistency across study sites. Since the sample sizes were smaller in the individual ADRC, we used univariate competing risk survival models, with only *APOE4* as an independent variable, to keep the number of parameters low and statistical power high. Of the 45 ADRC in NACC, 42 had autopsy data on at least one person. Of those 42 ADRC, 17 ADRC’s which had 165 or more study subjects with autopsy data (representing 81% of the total autopsied cases) had statistically significant SHR’s that were all in the same direction as our overall SHR finding (Fig. 2). The rest of the ADRCs had fewer than 164 subjects with autopsy data and/or missing other key data thereby yielded insufficient power to reach statistical significance for SHR in the competing risk models.

**Figure 3:**
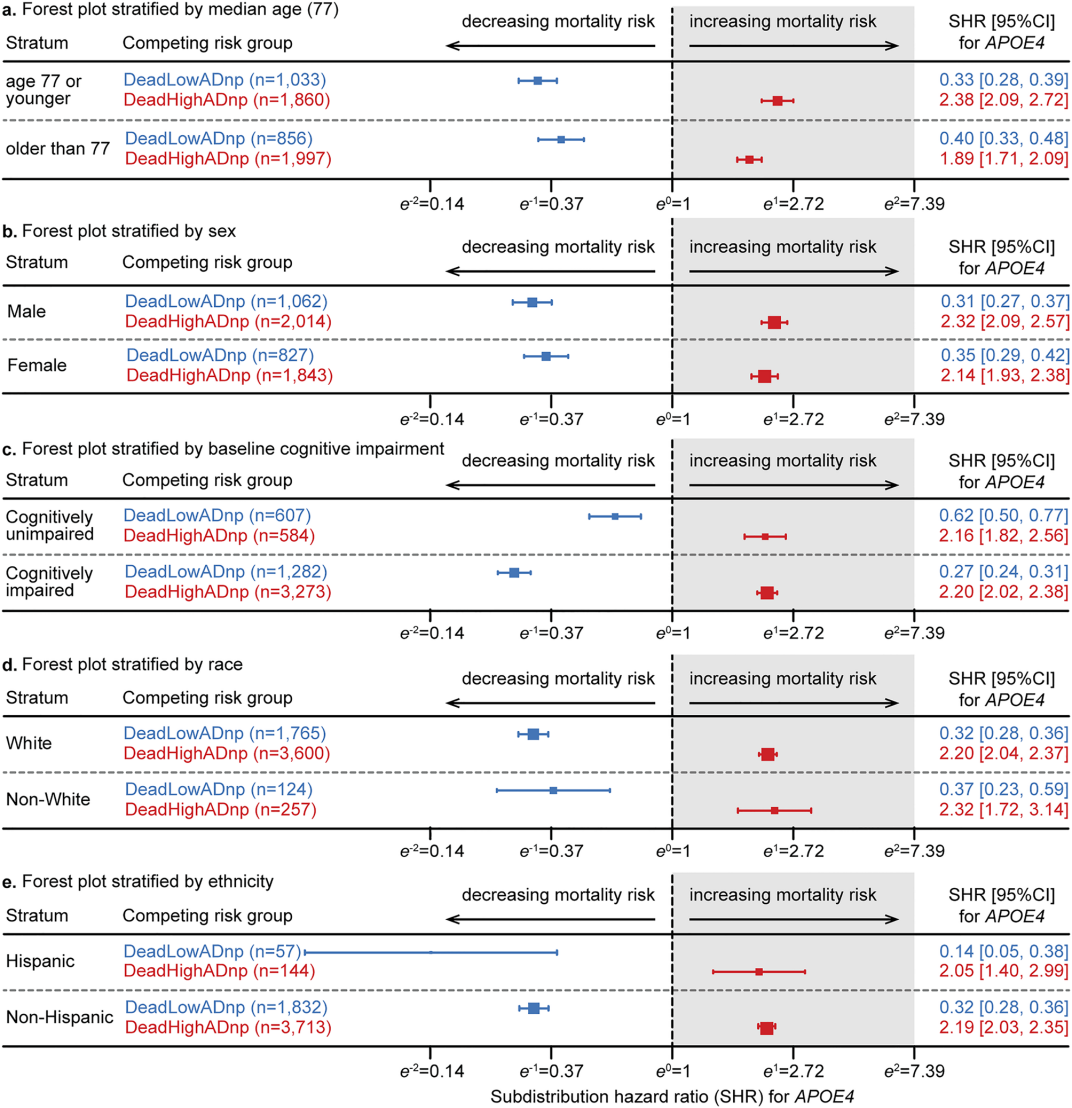
Forest plots of adjusted hazard ratios from multivariable Fine and Gray competing risk survival analyses within each stratum as stratified by baseline median age for autopsied subjects in panel (**a**), sex in panel (**b**), baseline cognitive impairment in panel (**c**), race in panel (**d**), and ethnicity in panel (**e**). Each multivariate model included the main explanatory variable *APOE4* and all covariates apart from the stratification variable. The models stratified by baseline median age, sex, and baseline cognitive impairment also included significant two-way interactions. The colored horizontal bars represent the estimated 95% confidence interval of the subdistribution hazard ratio (SHR) for *APOE4* on mortality risk, with the box in the middle at the estimated SHR scaled to the size of the sub-sample in each stratum. The dashed vertical line is at exp(β = 0) = 1, with decreasing mortality risk on the left and increasing mortality risk on the right. The SHR estimates for the competing risk group of death with low amounts of AD neuropathology (DeadLowADnp, shown in blue) are all to the left of the dashed vertical line, indicating decreased mortality risk was associated with *APOE4* for this competing risk group across all strata. The SHR estimates for the competing risk group of death with high amounts of AD neuropathology (DeadHighADnp, shown in red) are all to the right of the dashed vertical line, indicating increased mortality risk was associated with *APOE4* for this competing risk group across all strata.

The adjusted SHR for *APOE4* were further evaluated in multivariable competing risk survival models among each of the sub-groups or strata formed by several key variables: baseline age (split at the median), sex, baseline cognitive impairment, race, and ethnicity to check for stability of the *APOE4* HRs and consistency among these sub-groups. These models included *APOE4* as the main explanatory variable and all covariates apart from the stratifying variable. The fitted multivariable models among each of the sub-groups or strata split by baseline age, sex, and baseline cognitive impairment as well as the significant two-way interaction terms. The adjusted hazard ratios in the multivariable competing risk analysis within each stratum are consistent with the same general pattern. The 95% CI’s of the SHR’s were below one for the *APOE4* carriers in the DeadLowADnp group and above one for the *APOE4* carriers in the DeadHighADnp group across all sub-groups for each stratification variable (Fig. 3). In particular, when broken down further by race/ethnicity, the SHR’s were consistently below one in the DeadLowADnp group in the Black or African American, Asian, and non-White Hispanic sub-samples, although some had insufficient power to reach statistical significance.

## Discussion

In this study, we observed that *APOE4* carriers with low amounts of AD neuropathology at death had decreased mortality risk compared to non-carriers, but *APOE4* carriers with high amounts of AD neuropathology at death had increased mortality risk. Although self-identified race was the strongest predictor of consent for a brain autopsy, our main HRs of *APOE4* findings were consistent in non-White and Hispanic sub-samples. Similarly, all of the results from the univariate competing risk analyses in the individual Alzheimer’s Disease Research Centers were in the same direction as our main findings on HRs of *APOE4*. Importantly, the data from different centers were independent samples, so the consistency in the direction of the subdistribution hazard ratios across different centers indicates that our results were not swayed by strong effects from a few particular centers or sub-groups, or by an aberrant occurrence in the dataset.

Previous studies have reported *APOE4* carriers as a whole have increased all-cause mortality risk^25–28^. While our results for the competing risk group with high amounts of AD neuropathology agree with the mortality risk results of these previous studies, our observed association of decreased mortality risk in *APOE4* carriers with low amounts of AD neuropathology at death are contradictory to reported findings of the existing studies. Note that, these previous studies focused only on all-cause mortality and did not consider competing risks, which thereby led to severe bias in their findings on one of the two competing risk groups. Although many *APOE4* carriers develop AD and its related neuropathology, about 23% of non-demented elderly people globally are *APOE4* carriers^8^. Thus, our observed result of decreased mortality risk in *APOE4* carriers with low amounts of AD neuropathology could impact a sizeable portion of the general population.

At the population level, cognitive ageing and mortality risk associated with *APOE4* naturally depend on a trade-off with other leading causes of death in elderly people. Of particular importance are major age-related diseases such as cardiovascular diseases (e.g., heart attack, stroke, heart failure, coronary artery disease, ischemic heart disease) and cancers^10^. Numerous studies have shown that, in addition to an elevated risk for AD, *APOE4* carriers are at increased risk of cardiovascular diseases (CVD)^11–15^ and decreased risk for major types of cancers and many other diseases^19–21,32^. A number of studies have shown how these pleiotropic effects of *APOE4* can be beneficial to some *APOE4* carriers and detrimental to others^11,32–34^. Notably, in a prospective cohort study of 3,924 participants of the Framingham Heart Study Offspring cohort, Kulminski et al. observed that *APOE4* was antagonistically associated with onsets of CVD and cancer, where *APOE4* carriers were predisposed to an earlier onset of CVD and a delayed onset of cancer compared to the non-carriers^11^. In a phenome-wide association study of the UK Biobank data, Lumsden et al. found that *APOE4* carriers were at increased odds for AD and ischemic heart disease, and decreased odds for gallbladder disease and liver disease compared to non-carriers^32^. Opposing effects of *APOE4* on neurodegeneration were observed in a mouse model by Hudry et al., where they hypothesized that *APOE4* may be neurotoxic during early stages of amyloid deposition in the development of AD, but may be neuroprotective in later stages of ageing^33^. These reported pleotropic effects of *APOE4* could have contributed in several ways to the differences in mortality risk we observed among *APOE4* carriers with low versus high amounts AD neuropathology at death.

There are many studies which have found that *APOE4* carriers have elevated risk for CVD (either early or late onset)^11–15^, which has been the number-one leading cause of death globally for decades^9^. In the NACC dataset, *APOE4* carriers have an elevated risk of clinician reported myocardial infarction among people with a concurrent AD diagnosis (n = 4,569) but not in those without an AD diagnosis (n = 13,009) at the baseline assessment of clinician reported comorbidities in the past 12 months. These results indicate that among *APOE4* carriers in the competing risk group with high amounts of AD neuropathology, there is higher risk of CVD-related death, which directly supports our finding of elevated mortality risk in this group. The two-way interaction between *APOE4* and sex that we observed in subjects with high amounts of AD neuropathology may also be related to death with CVD. Since men and *APOE4* carriers are at greater risk for CVD-related death than women and non-carriers of *APOE4*^9,15^, the combined risk for men who are *APOE4* carriers could make that sub-group particularly vulnerable to CVD-related death.

The association of *APOE4* with early-onset CVD as a cause of death^11^ could also be a contributing factor to our findings on mortality risk for *APOE4* carriers with low AD neuropathology. First, people with early-onset CVD would be more likely to die, or be too unhealthy, at a younger age to participate in an Alzheimer’s disease study at NACC. This would decrease the observed hazard of death with early-onset CVD among NACC participants, particularly among *APOE4* carriers who are more susceptible to early-onset CVD. Second, people with a non-fatal CVD event, or those with a family history of CVD, would be more likely than not to be taking medication or preventative measures to treat or delay the onset of CVD. This may not only decrease the hazard of death with early-onset CVD among NACC participants, but these medications and preventative measures may also provide some protection from cognitive decline or accumulation of AD neuropathology according to multiple published studies^35–38^. Thus, *APOE4* carriers in the competing risk group with low amounts of AD neuropathology may have lower risk of CVD-related death than the general population which could have contributed to the decreased mortality risk for people in this group.

*APOE4* carriers with low AD neuropathology may also benefit from potential protective effects against cancer, which is the second leading cause of death^10^. In addition to the trade-off described by Kulminski et al. with *APOE4* postponing cancers to older ages^11^, other studies have also reported that *APOE4* carriers have lowered mortality risk for some common cancers including melanoma^19^, colon cancer^20^, and colorectal neoplasm^21^. These results are concurrent with the NACC data where *APOE4* was associated with being cancer free at the baseline assessment of clinician reported diagnosis of cancer in the past 12 months. Considering these results, one could expect that among those *APOE4* carriers with low amounts of AD neuropathology, the risk for cancer is also reduced compared with the general population, and therefore contributes to decreased mortality risk in this group.

In addition to the trade-off of risk with age-related diseases, the theory of antagonistic pleiotropy might also be considered in relation to mortality and *APOE4*^39,40^. In the theory of antagonistic pleiotropy, genes that are related to detrimental effects in ageing persist in the population because they contribute to fertility and/or have beneficial survival effects in early life. *APOE4* is believed to promote inflammation as an innate immune response to infections in early life, but this beneficial inflammation process may become problematic later in life as people become more prone to age-related diseases at older ages. A number of studies have linked *APOE4* to enhanced fertility^40,41^ and better outcomes related to infectious diseases^42–45^, particularly in early life. It is possible that these same protective mechanisms continue to protect *APOE4* carriers if they do not become susceptible to developing age-related diseases and AD neuropathology. This would contribute to the lowered mortality risk among this group of *APOE4* carriers with low AD neuropathology.

Reported interactions between *APOE4* and other genes which provide protection against shortened lifespans^46^ or reduce susceptibility to AD neuropathology^47,48^, present interesting opportunities for future explorations of potential mechanisms which could lead to decreased mortality risk among *APOE4* carriers with low amounts of AD neuropathology and abnormal cognitive ageing. Lin et. al. observed interactions between *APOE4* and Wnt signaling genes, where there was a pro-longevity effect of rare coding variants in the Wnt signaling pathway for *APOE4* carriers^46^. A study by Belloy et al. reported that interactions between *APOE4* and *Klotho* (a longevity gene) were associated with reduced AD risk and amyloid pathology burden in a subset of *APOE4* carriers^47^. However, Chen et al. indicated that the interaction between *APOE4* and *Klotho* may not only provide protection of reduced AD risk and slowed progression in the early stages of the disease for *APOE4* carriers, but also provide significant protection of slowed cognitive decline for non-carriers in later disease stages^49^. In a mouse model, Tachibana et al. observed an interaction between *APOE4* and *LRP1* (a protein coding gene) where mice with *LRP1* and *APOE4* had increased amyloid pathology, but *LRP1*-knockout mice with *APOE4* did not have increased amyloid pathology^48^. It is possible that interactions of *APOE4* with some combination of these other genes could provide protection against cognitive decline and the accumulation of AD neuropathology, thereby reducing mortality risk among *APOE4* carriers. Genetic data from the National Institute on Ageing Genetics of Alzheimer's Disease Data Storage Site for the NACC cohort is a valuable resource for future studies to provide insight into potential genes which interact with *APOE4* that are associated with the results of our study.

The process of abnormal cognitive ageing associated with the excess accumulation of AD neuropathology has devastating effects on the quality of life for patients and has a detrimental impact on mortality risk and lifespans. Considering that *APOE4* is not rare, our findings on differential mortality risks in *APOE4* carriers are relevant to both lifespans and quality of life for a sizable portion of the general population. Moreover, our findings open up interesting opportunities for future studies on factors which could protect *APOE4* carriers and other vulnerable sub-groups against abnormal cognitive ageing and prolong their lives. Given that there is no cure nor effective treatment for abnormal cognitive ageing from the accumulation of AD neuropathology, it is of interest to investigate potential alterable lifestyle measures that might reduce risk of late-onset AD among vulnerable subpopulations. Our findings have significant implications on the possibility of reducing mortality risk through preventing the accumulation of AD neuropathology among *APOE4* carriers, and possibly other vulnerable subpopulations as well. Preventive lifestyle measures for CVD (e.g., smoking abstinence or cessation, regular exercise, healthy diet, improved sleep) have been shown to have beneficial effects on cognition and other functions of the brain^50–53^. Some commonly used dietary supplements such as omega-3 and fish oil products have been shown to have protective effects for CVD, as well as AD and its related neuropathology^38^. There are many studies of commonly used FDA-approved medication for hypertension^35^, heart disease, diabetes (e.g., metformin)^37^, that have shown benefits for reduced risk of AD neuropathology and cognitive decline. We believe that the two-way interaction we observed between *APOE4* and baseline cognitive impairment among people with low amounts of AD neuropathology may be influenced by these types of preventative measures. Specifically, *APOE4* carriers are more likely to have a family history of AD and/or CVD which could make them more proactive about changes in their cognition and overall health. Altogether, it is warranted to further study and optimize these preventative measures, dietary supplements, and medications intended to treat or prevent CVD, or other age-related diseases, so that some combinations may become effective in protecting against cognitive decline, reducing mortality risk, and prolonging health span and lifespans in vulnerable sub-groups.

A major limitation of our study is that the NACC data is not a random sample with the majority of enrollees identifying as non-Hispanic White. Thus, our findings may not extend beyond non-Hispanic White populations. Autopsies were performed in a non-random sub-sample of all the dead subjects, and we account for this potential participation bias by using the autopsy propensity scores. However, there may also be further bias related to the high proportion of NACC enrollees with *APOE4* or other perceived AD risk factors. Conversely, *APOE4* carriers who may be less susceptible to developing AD neuropathology (e.g., those without a family history of AD) could be underrepresented in the NACC data, since they may not be aware of carrying *APOE4*. Further research on characterizing *APOE4* and other factors associated with abnormal cognitive ageing, and developing needed preventive measures are warranted.

## Methods

### Sample

The data for this study came from the National Alzheimer’s Coordinating Center (NACC) data repository which is composed of data submitted from 45 past and presently active NIA Alzheimer’s Disease Research Centers (ADRC) from across the US. All studies and all experimental protocols from every ADRC were approved by their respective Institutional Review Board prior to study initiation. Written informed consent was obtained for all study participants at each ADRC. All studies and methods at all participating ADRC’s were conducted in accordance with the principles expressed in the Declaration of Helsinki. In short, local Institutional Review Boards (IRBs) approve ADRC research activities. NACC data are de-identified, and research involving the NACC database is approved by the University of Washington IRB.

As of March 2023, the NACC database included 45,998 individuals recruited since 2005. All of our analysis excluded individuals who were missing *APOE* genotyping (n = 11,565). People who were dead but did not have a brain autopsy (n = 3,894) were compared to those that had a brain autopsy (n = 5,858) in developing the autopsy propensity model. People who were dead but did not have a brain autopsy used for developing the propensity score model, and 112 individuals who had a brain autopsy but were missing key data for group characterization, were excluded from the competing risk survival models. The remaining 5,746 individuals with the key brain autopsy data were divided into two competing risk groups: death with low amounts of AD neuropathology (DeadLowADnp, n = 1,889) and death with high amounts of AD neuropathology (DeadHighADnp, n = 3,857). Individuals without reported death (n = 24,681) were also included as censored at their last clinical visit for the competing risk survival models.

### Autopsy Assessment

Alzheimer’s disease neuropathology assessed at autopsy in the NACC data before 2014 included neurofibrillary tangles (Braak stages) and neuritic plaques (CERAD scores)^30,31^. At that time, the NACC data also included a primary neuropathological diagnosis (e.g., AD, Lewy Body dementia, vascular dementia). In 2014, the autopsy diagnosis was replaced by the ABC score, which was added to the NACC data along with the assessment of amyloid plaques (Thal phases)^30^. The ABC score provides a standardized quantification based on the Thal phases (score A), Braak stages (score B) and CERAD scores (score C)^31^. In this study, both the earlier method and the ABC scores were used to quantify the general amounts of AD neuropathology at death as either low (DeadLowADnp) or high (DeadHighADnp). The DeadLowADnp competing risk group included individuals who died with lower amounts of Alzheimer’s neuropathology, characterized by an autopsy assessed ABC score of none or low, a non-AD primary autopsy diagnosis, a B score of 0 or 1 but missing A and/or C score, or an A score of 0 or 1 along with a C score of 0 or 1 but missing B score. The DeadHighADnp competing risk group included individuals who died with higher amounts of Alzheimer’s neuropathology, characterized by an autopsy assessed ABC score of intermediate or high, an AD primary autopsy diagnosis, a B and C score of 2 or more but missing A score, or a B and A score of 2 or more but missing C score. We summarized the demographic and clinical characteristics of the competing risk groups in Table 1 and reported the *APOE* allele distributions in Table 2.

### Statistical analysis—autopsy propensity model

A logistic regression model predicting autopsy participation among all dead subjects was formed to derive an autopsy propensity score. Forward/backward selection with cross-validation (R package “rms” v6.3–0) was used for variable selection from the demographic and clinical variables. We tested *APOE4* as a predictor both univariately and in the final propensity model with the selected covariates, to check if it appeared to be randomly distributed between people who did and did not have autopsies. The predicted autopsy propensity scores from the final model were generated for all individuals in the study (living and dead) and divided into *J* = 20 discrete percentile categories. The stratified competing risk survival models included these 20 autopsy propensity score categories as model strata.

### Statistical analysis—competing risks survival models

Age at death was used as the time to the outcome events (death with low vs. death with high AD neuropathology) and age at the last clinical evaluation was treated as the censoring time for living individuals. *APOE4* (with non-carriers as the reference group) was the main explanatory variable in all competing risk survival models as it was the main effect of interest. Baseline age was included as a covariate and all other demographic and clinical covariates were selected with the forward/backward variable selection method. We further tested two-way and three-way interactions between all covariates. Backward selection was used in the model of the CSHR to select significant interactions between covariates. The selected interactions were then tested in the Fine and Gray model of the SHR. Only interactions that were significant in both the CSHR and SHR were retained in the analysis reported in Table 3.

We used the Fine and Gray competing risk survival model^22,23^ to estimate the subdistribution hazard ratio (SHR) and to calculate the cumulative incidence function (CIF). We also fitted a cause-specific hazards (CSH) competing risk survival model^22,24^ to estimate the cause-specific hazard ratio (CSHR). With the *J* = 20 autopsy propensity score percentile categories as model strata and the *K* = 2 competing risk groups (DeadLowADnp and DeadHighADnp), both models have the following proportional-hazard form:

1 $${h}_{k}\left({t}_{ijk},{{\varvec{X}}}_{ijk}\right)= {h}_{0jk}\left({t}_{ijk}\right)\mathrm{ exp}\left({\widehat{{\varvec{\beta}}}\mathbf{^{\prime}}}_{k}{{\varvec{X}}}_{ijk}\right)$$

where, for each subject *i* in stratum *j* and competing risk group* k*, *t* is age at death (or age at the last clinical visit for censored subjects), *h*_*0*_*(t)* is the baseline hazard, ***X*** represents a vector of covariates including *APOE4* (presence or absence) and the selected covariates (including main effects and interaction terms), and ${\widehat{{\varvec{\beta}}}\mathbf{^{\prime}}}_{k}$ represents the corresponding vector of estimated regression coefficients (which are fixed across* j* strata as in typical stratified Cox PH model settings) for each competing risk group *k*.^54,55^

We used both the Fine and Gray and the CSH competing risk survival models because they estimate the hazard rates differently but both are commonly reported in literature. The hazard rate equation for the cause-specific hazard function from the CSH model is:

2 $${h}_{{k}_{C}}(t) = \underset{\mathit{\delta t}\to 0}{\mathrm{lim}}\left\{\frac{P(t\le T<t+\delta t, K=k| T\ge t)}{\delta t}\right\}$$

where ${h}_{{k}_{C}}(t)$, is the instantaneous (i.e. $\delta t$) risk of dying if a subject is in the competing risk group *k* given that the subject is still alive by age *t*. Thus, in the CSH competing risk survival model, all subjects not in a particular competing risk group are counted as censored observations when estimating the CSHR in that particular competing risk group. The hazard rate equation for the subdistribution hazard from the Fine and Gray model is:

3 $${h}_{{k}_{S}}(t) = \underset{\mathit{\delta t}\to 0}{\mathrm{lim}}\left\{\frac{P(t\le T<t+\delta t, K=k| T>t\; or\; (K\ne k\ \&\ T\le t))}{\delta t}\right\}$$

where ${h}_{{k}_{S}}(t)$ is the instantaneous risk of dying if a subject is in the competing risk group *k* given that the subject is still alive by age *t* or is in a different competing risk group and has already died. Thus, in the Fine and Gray competing risk survival model, the SHR estimates the effect of the covariates in the presence of the other competing risks, rather than including them as censored observations as in the CSHR estimation. The Fine and Gray model was used to calculate the associated cumulative incidence function (CIF) and the corresponding 95% confidence intervals shown in Fig. 1.

The R packages “survival” (v3.4–0), “cmprisk” (v2.2–11), “crrSC” (v1.1.2), and “timereg” (v2.0.2) were used to generate CSHR (unstratified and stratified), unstratified SHR, stratified SHR, and CIF plots (Fig. 1), respectively. The models were also assessed without including censored subjects, propensity-score stratification, or adjusting for covariates to check for robustness of the results. Within each individual ADRC, a univariate Fine and Gray competing risk survival model was used to assess mortality risk for *APOE4* carriers to check for stability and consistency across all ADRCs as shown in Fig. 2. Also, the adjusted SHR for *APOE4* were further evaluated in multivariable competing risk survival models among each of the sub-groups or strata formed by several key variables: baseline age (split at the median), sex, baseline cognitive impairment, race, and ethnicity to check for stability and consistency of the *APOE4* HRs among these sub-groups (Fig. 3). There is a small portion of study subjects with information available on some clinician diagnosed comorbidity (e.g. CVD, cancer) in the NACC data. We informally reported the relative abundance of the comorbidity among the subgroup of *APOE4* carriers and non-carriers with available comorbidity data. We did not conduct formal statistical comparison or calculate p-values due to missing data in majority of study subjects.

## Data Availability

All data used in this study is available from the National Alzheimer’s Coordinating Center, at alz.washington.edu, to qualified researchers on the condition of signing the NACC Data Use Agreement. The first author had full access to all the data in the study and takes responsibility for the integrity of the data and the accuracy of the data analysis.
